# National Surveillance on Vancomycin-Resistant *Enterococcus faecium* in Taiwan: Emergence and Widespread of ST414 and a Tn*1546*-Like Element with Simultaneous Insertion of IS*1251*-like and IS*1678*

**DOI:** 10.1371/journal.pone.0115555

**Published:** 2014-12-30

**Authors:** An-Jing Kuo, Lin-Hui Su, Jwu-Ching Shu, Jann-Tay Wang, Jen-Hsien Wang, Chang-Phone Fung, Ju-Hsin Chia, Jang-Jih Lu, Tsu-Lan Wu

**Affiliations:** 1 Department of Laboratory Medicine, Chang Gung Memorial Hospital, Linkou, Taoyuan, Taiwan, ROC; 2 Department of Medical Biotechnology and Laboratory Science, College of Medicine, Chang Gung University, Taoyuan, Taiwan, ROC; 3 Research Center for Pathogenic Bacteria, Chang Gung University, Taoyuan, Taiwan, ROC; 4 Division of Infectious Diseases, Department of Medicine, National Taiwan University Hospital, Taipei, Taiwan, ROC; 5 Division of Infectious Diseases, Department of Internal Medicine, China Medical University Hospital, Taichung, Taiwan, ROC; 6 Section of Infectious Diseases, Department of Medicine, Taipei Veterans General Hospital, National Yan-Ming University, Taipei, Taiwan, ROC; The University of Hong Kong, Hong Kong

## Abstract

Cases of bacteremia caused by vancomycin-resistant *E. faecium* (VRE-fm) increased significantly in Taiwan. The present multicenter surveillance study was performed to reveal the associated epidemiological characteristics. In 2012, 134 non-repetitive VRE-fm isolates were prospectively collected from 12 hospitals in Taiwan. Antimicrobial susceptibility, pulsed-field gel electrophoresis (PFGE), multilocus sequence typing (MLST), and analysis of *van* genes and Tn*1546* structures were investigated. Two isolates carried *van*B genes, while all the remaining isolates carried *van*A genes. Three isolates demonstrated a specific *van*A genotype *- van*B phenotype. Nine (6.7%) isolates demonstrated tigecycline resistance, and all were susceptible to daptomycin and linezolid. Molecular typing revealed 58 pulsotypes and 13 sequence types (STs), all belonged to three major lineages 17, 18, and 78. The most frequent STs were ST17 (n = 48, 35.8%), ST414 (n = 22, 16.4%), and ST78 (n = 16, 11.9%). Among the *van*A harboring isolates, eight structure types of the Tn*1546*-like element were demonstrated. Type I (a partial deletion in the *orf1* and insertion of IS*1251*-like between the *vanS* - *vanH* genes) and Type II (Type I with an additional insertion of IS*1678* between *orf2* - *vanS* genes) were the most predominant, consisted of 60 (45.5%) and 62 (47.0%) isolates, respectively. The increase of VRE-fm bacteremia in Taiwan may be associated with the inter- and intra-hospital spread of some major STs and horizontal transfer of *van*A genes mostly carried on two efficient Tn*1546-*like elements. The prevailing ST414 and widespread of the Type II Tn*1546*-like elements are an emerging problem that requires continuous monitoring.

## Introduction

Enterococci are a part of the normal intestinal flora in human and animals. They may also colonize many body sites of healthy individuals and cause opportunistic infection in immunocompromised patients. These organisms are also a common cause of hospital-acquired infection (HAI) and are associated with a substantial proportion of bloodstream and urinary tract infections. Enterococci are intrinsically resistant to a variety of antibiotics. Vancomycin is usually required for treatment, especially for invasive infections. Therefore, treatment of infections caused by vancomycin-resistant enterococci (VRE) may be difficult.

Vancomycin resistance in enterococci, or to be specific in *Enterococcus faecium* (VRE-fm), was firstly reported in 1986 in the UK and France [1], [2]. Until now, VRE have been reported globally and caused HAI in the North American [3], [4], Europe [5], and Asia [6]–[8]. The prevalence of VRE is increasing, especially in *E. faecium* [9]. A recent report revealed that the frequency of VRE-fm was high in the North America (76%), Latin America (48.4%), Europe (31.5%), and the Asia/Pacific region (14.1%) [5]. The development of vancomycin resistance involved the acquisition of *van* genes. At present, nine genotypes of *van* genes (*van*A, *van*B, *van*C, *van*D, *van*E, *van*G, *van*L, *van*M, *van*N) have been reported, with *van*A and *van*B being the most predominant [10]. The *van*A operon is usually carried by a Tn*3*-type transposon, Tn*1546*, that is consisted of five genes (*vanHAXYZ*) involving glycopeptide resistance, two regulatory genes (*vanRS*), and transposase (*orf1*) and resolvase (*orf2*) regions [10]. Several genetic variations, including the presence of insertion sequences or deletions in nonessential genes and/or intergenic regions, have been reported in Tn*1546* [8], [11], [12].

In Taiwan, VRE was firstly reported in 1996 [13]. According to the reports from the Centers for Disease Control, Taiwan, and others from Taiwan, a significant increase of VRE-fm infection has been noted since 2008, especially in the northern and central Taiwan [14]–[16]. High selective pressure from antimicrobial usage was found to have contributed to the increase of VRE-fm infections [17]–[19]. In 2012, a nationwide, multicenter surveillance study was prospectively conducted at 12 hospitals in Taiwan. The genotypes (pulsotypes, sequence types) of VRE-fm clinical isolates and *van* gene analysis, including structure analysis of *van*A/Tn*1546*, were investigated and reported herein.

## Materials and Methods

### Ethics statement

The present study aimed to characterize vancomycin-resistant *E. faecium* isolates using molecular methods. All isolates studied were prospectively collected from 12 hospitals in Taiwan. Clinical information of the patients was not required in this study. Because all microbial cultures were ordered by physicians due to clinical necessity and none was collected purposely for this study, patients’ informed consents were not required and therefore were not collected.

### Hospital settings and bacteria

Between January and December 2012, a total of 134 non-repetitive VRE-fm blood isolates were collected prospectively from 12 hospitals in Taiwan. A total of 9 medical centers (each consisted of 1000–4700 beds, respectively) and 3 regional hospitals (each with 500–1100 beds, respectively) distributed among 4 geographic regions of Taiwan were included in the study. These included the Chang Gung Memorial Hospital, Linkou (N_1_), National Taiwan University Hospital (N_2_), Taipei Veterans General Hospital (N_3_), Tri-Service General Hospital (N_4_), and Chang Gung Memorial Hospital, Keelung (N_5_) in the north; the China Medical University Hospital (C_1_) in the central region; the Kaohsiung Medical University Hospital (S_1_), Kaohsiung Municipal Hsiaokang Hospital (S_2_), Kaohsiung Chang Gung Memorial Hospital (S_3_), Chang Gung Memorial Hospital, Chiayi (S_4_), Chi Mei Medical Center (S_5_) in the south; and the Buddhist Tzu Chi General Hospital (E_1_) in the east. All VRE-fm isolates were sent to the central laboratory at the Chang Gung Memorial Hospital, Linkou (N_1_) for the subsequent experiments. The identity of the enterococci was confirmed with the use of a commercial Rapid 32 Strep Kit (BioMerieux, Hazelwood, France) in the central laboratory.

### Susceptibility testing

Antibiotic susceptibility testing of ampicillin, ciprofloxacin and high-level gentamicin (Becton Dickinson, Franklin Lakes, NJ, USA) was performed by a disc diffusion method. Minimum inhibitory concentrations (MICs) of vancomycin, teicoplanin, linezolid, daptomycin and tigecycline were evaluated with E-test strips (AB Biodisk, Solna, Sweden). The results were interpreted according to the recommendations by the Clinical and Laboratory Standards Institute [20]. The susceptibility for tigecycline was defined based on the European Committee on Antimicrobial Susceptibility Testing criteria (MIC≤0.25 µg/mL) [21]. *Staphylococcus aureus* ATCC29213 and *Enterococcus faecalis* ATCC29212 were used as control strains.

### Detection of the vancomycin resistance genes

DNA of the isolates was extracted by using the QIAamp DNA Mini Kit (Qiagen, Hilden, Germany). The vancomycin resistance genes, *van*A, *van*B, *van*C1 and *van*C2/C3, were detected using a multiplex PCR as previously described [22].

### Multilocus sequencing type (MLST) and pulsed-field gel electrophoresis (PFGE)

MLST was performed according the method described previously [23]. The amplicons were purified using Microcon PCR Centrifugal Filter Devices (Millipore Corporation) and sequenced using an ABI 3100-Avant Genetic Analyzer (Applied Biosystems). The sequence type (ST) was determined through the comparison with the MLST database on the public domain (http://efaecium.mlst.net). Clustering analysis of the STs was performed by the eBURST program through the web site. PFGE was performed by a previously described method [24]. The resulting patterns were analyzed by BioNumerics (version 6.5, Applied Maths, Austin, Texas). PFGE patterns with more than 80% similarity were considered as closely related and categorized into the same pulsotypes.

### Molecular analysis of the Tn*1546*-like elements

The presence and the configuration of the Tn*1546*-like elements were analyzed using a published PCR overlapping technique with 10 pairs of primers [25]. The Tn*1546* prototype (GenBank accession no. M97297) was used as the reference. Amplicons with unexpected fragment sizes were subjected to further sequence analysis.

### Statistical analysis

Statistical analysis was performed by the Chi-square test or Fisher’s extract test when appropriated. A value of *p*<0.05 was considered statistically significant.

## Results

### Antimicrobial susceptibility and *van* gene analysis

A total of 134 VRE-fm isolates were confirmed and enrolled in the present study. The majority (n = 132, 98.5%) of the isolates carried a *van*A gene. Vancomycin MICs were high (>256 µg/mL) among the isolates with the *van*A genes, but various MICs (4 - >256 µg/mL) were observed for teicoplanin. Among them, 3 isolates demonstrated a specific *van*A genotype-vanB phenotype [26] with the teicoplanin MICs ranged between 4–8 µg/mL. Another 2 isolates carried the *van*B gene were associated with a much lower MICs (vancomycin, 8 µg/mL; teicoplanin, 1 µg/mL).

In addition to glycopeptides resistant, the VRE-fm isolates were all resistant to ampicillin and ciprofloxacin. The resistance rate to high-level gentamicin was 48.2%. However, resistance to the newer antibiotics remained low, all 134 VRE-fm isolates were susceptible to Daptomycin (MIC 0.06–4 µg/mL) and Linezolid (MIC 0.25–2 µg/mL). Only 9 (6.7%) isolates demonstrated tigecycline (MIC 0.16–12 µg/mL) resistance.

### MLST and PFGE analysis

**Figure 1:**
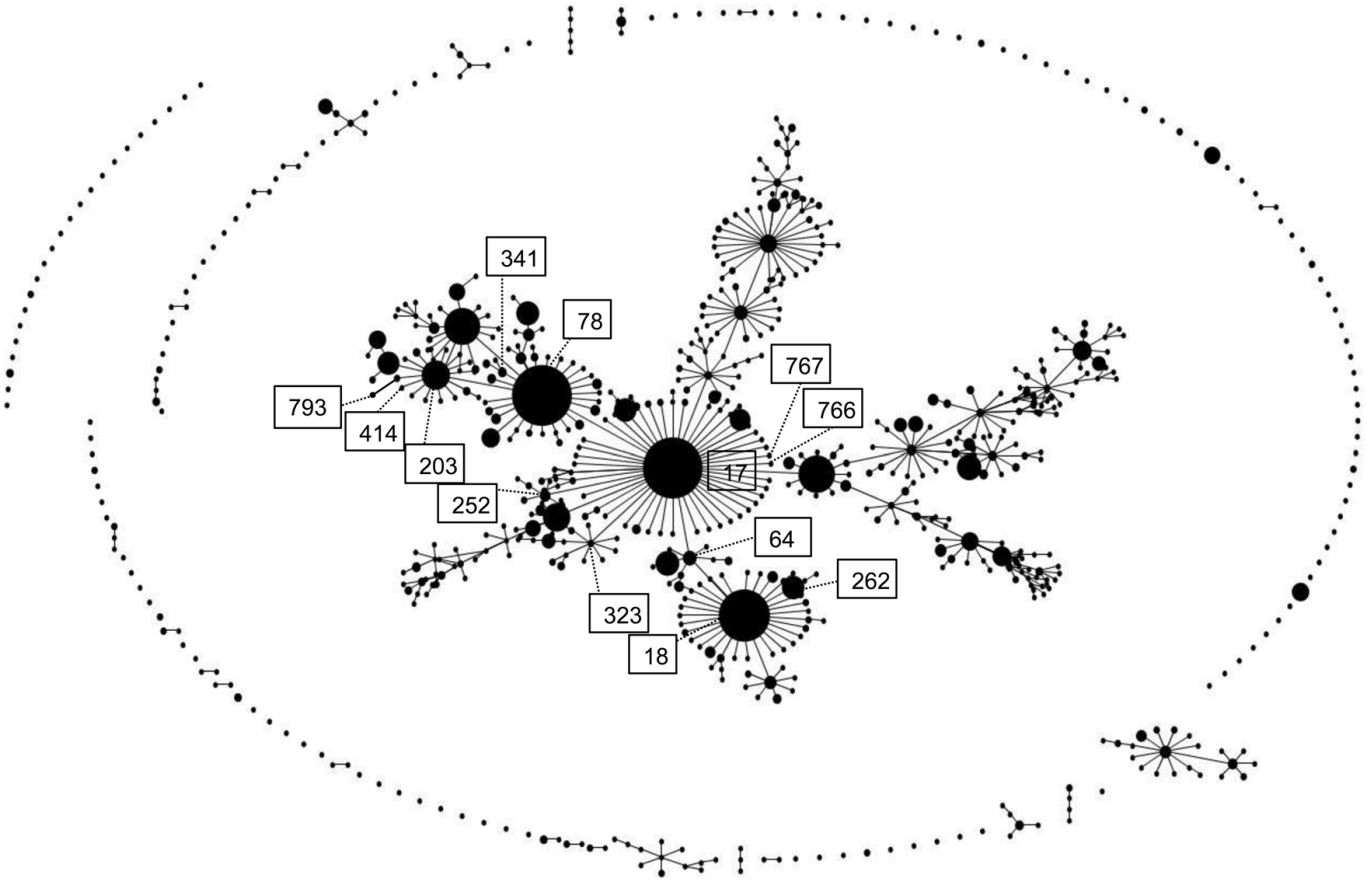
eBURST analysis of the 134 VRE isolates in the present study and those published MLST database. Each ST is presented as a dot. The size of each dot corresponds to the number of isolates in the ST. The 13 STs found in this study are indicated in boxed numbers.

Through MLST analysis, 13 sequence types (STs) were identified from the 134 VRE-fm isolates (Fig. 1). Three STs (ST766, ST767 and ST793) were newly identified in the present study. The most predominant STs were ST17 (n = 48, 35.8%), ST414 (n = 22, 16.4%), ST78 (n = 16, 11.9%), ST341 (n = 13, 9.7%), and ST18 (n = 11, 8.2%). The 2 isolates carried the *van*B gene belonged to ST64, and the 3 isolates demonstrated the specific *van*A genotype-vanB phenotype were all ST78. In contrast, the 9 tigecycline-resistant isolates were distributed among 5 STs. Comparison of the 13 STs identified in the present study with those published STs was further performed. As shown in Fig. 1, eBURST analysis showed that all VRE-fm isolates belonged to the clonal complex 17 (CC17). Base on the suggestion from a recent publication on the population structure analysis of hospital *E. faecium* isolates [27], the isolates were subgrouped into three lineages: lineage 17 (5STs: ST17, ST252, ST323, ST766 and ST767, n = 58, 43.3%), lineage 18 (3 STs : ST18, ST64, ST262, n = 22, 16.4%), and lineage 78 (5 STs : ST78, ST203, ST341, ST793 and ST414, n = 54, 40.3%).

**Figure 2:**
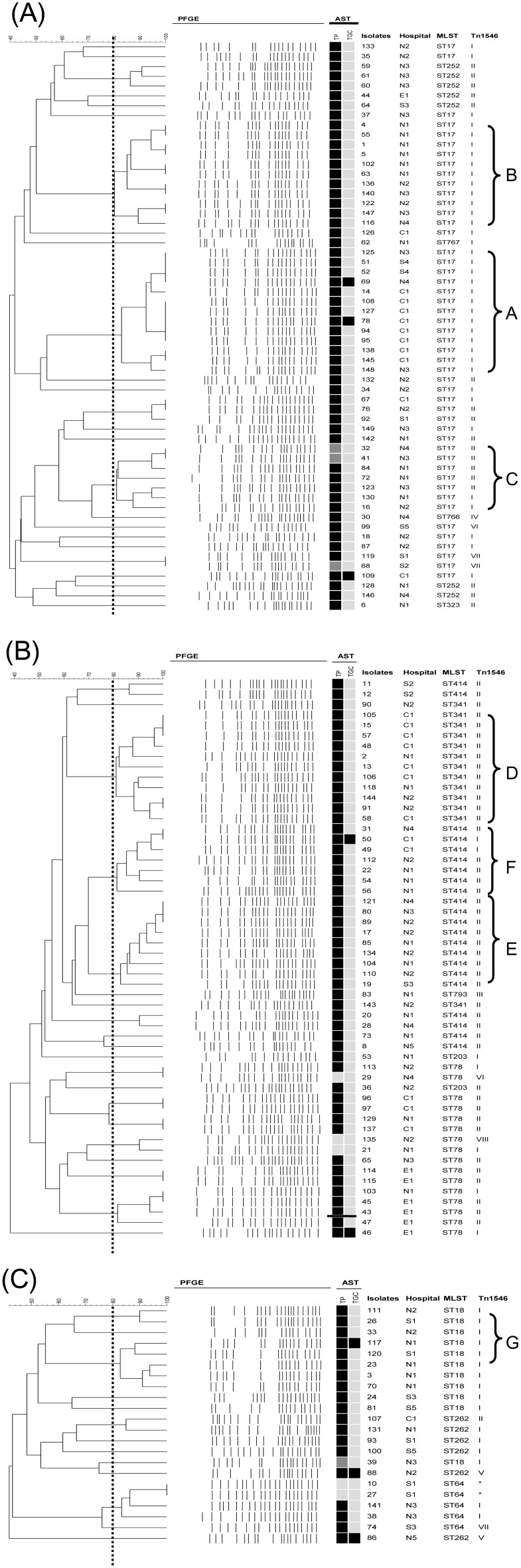
Pulsed-field gel electrophoresis (PFGE) dendrogram in (A) lineage 17, (B) lineage 78, and (C) lineage 18. Detailed information on the results of antimicrobial susceptibility testing (AST), hospitals, multilocus sequence types (MLST), and Tn*1546* structure types (Tn*1546*) are listed, respectively, for each isolate. Pulsotypes A-G are clustered based on 80% similarity of the PFGE patterns. TP and TGC indicate teicoplanin and tigecycline. The squares indicate antimicrobial susceptibility results: black, resistant; gray, intermediate, and pale gray, susceptible. Configurations of the structure types of Tn*1546* are as described in Fig. 2. Two *vanB*-carrying isolates are indicated with an asteroid mark (*).

PFGE analysis revealed 58 pulsotypes: 25 were found in the lineage17 (Fig. 2A) with 3 major pulsotypes, A-C, belonging to ST17; 20 were found in the lineage 78 (Fig. 2B) with another 3 major pulsotypes belonging to ST341 (pulsotype D) and ST414 (pulsotypes E and F); and the remaining 13 were found in the lineage18 (Fig. 2C), with one major pulsotype G belonging to ST18. Small clusters of a few particular genotypes were noted in some hospitals: hospital C_1_, ST17/pulsotype A (n = 8) and ST341/pulsotype D (n = 7); hospital N_1_, ST17/pulsotype B (n = 6) and ST18/pulsotype G (n = 4); and hospital N_2_, ST414/pulsotype E (n = 4). Isolates belonged to pulsotypes A–G could be found among 2–5 hospitals.

### Characterization of Tn*1546*-like elements

**Figure 3:**
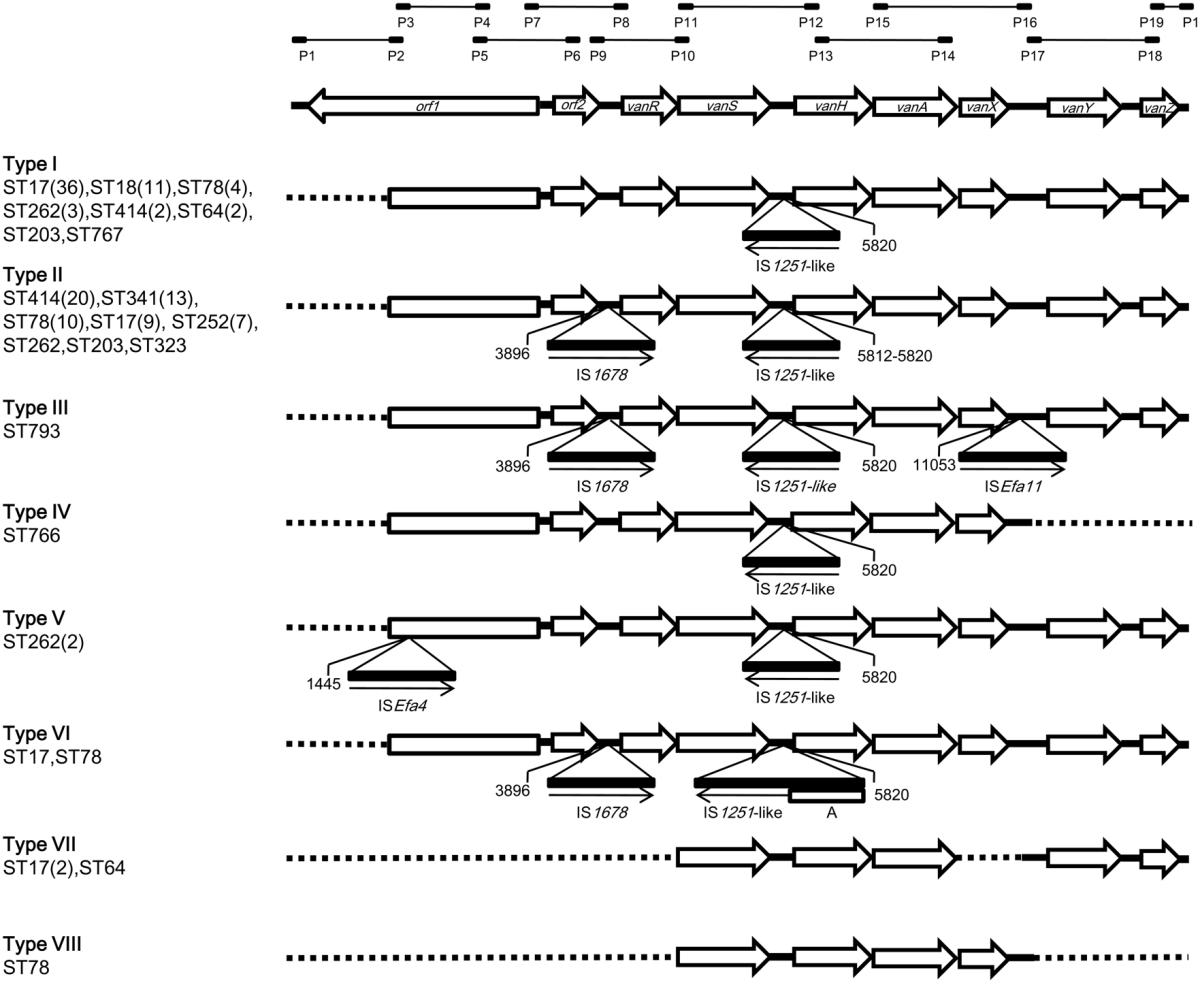
Genetic maps of Tn*1546* structures found among the 132 *van*A-containing VRE isolates studied. Primers (P1– P19) used were based on the scheme described by Arthur et al. [25]. The positions of genes and open reading frames and the direction of transcription are depicted with open arrows. IS elements are indicated by triangles. The positions of the nucleotide upstream to the IS insertion sites are depicted. Arrows under the triangles indicate the transcriptional orientations of the inserted IS elements. Dotted lines indicate no amplicon by the corresponding primers. A, an extra DNA sequence inserted without further characterization. STs carrying those structure types are indicated to the left of the maps, respectively. Numbers of isolates are indicated in parentheses if more than one was found.

Genetic structures of the Tn*1546*-like elements were further analyzed among the 132 *van*A-carried VRE-fm isolates. Eight different Tn*1546* structure types were detected among the VRE isolates studied herein (Fig. 3). The prototype of Tn*1546* (GenBank accession no. M97297) was not found in any of the isolates studied. None of the isolates produced positive results by using the first PCR primer pair (P1-P2), while all isolates produced amplicons of the expected size when the 7^th^ primer pair (P13-P14) was used. Diverse results were found with the use of the other 8 primer pairs, and accordingly 8 structure types were identified.

Types I and II were the most predominant, consisted of 60 (45.5%) and 62 (47.0%) isolates, respectively. Type I was characterized by the insertion of an IS*1251*-like element at nt 5820, the intergenic region between the *vanS* and *vanH* genes. DNA sequences of the IS*1251*-like element were identical to those published in the GenBank database with accession number AF148130. Eight STs (ST17, ST18, ST64, ST78, ST203, ST262, ST414, ST767) were found in Type I. Type II was similar to Type I except that an additional insertion of IS*1678* at nt 3896, the intergenic region of *orf2*-*vanS* genes, was noted. One more difference was found in one Type II isolate in which the insertion of IS*1251*-like was found at nt 5812. Type II also consisted of 8 STs: ST17, ST78, ST203, ST252, ST262, ST323, ST341 and ST414. Among them, 4 STs were solely associated with one single structure type of Tn*1546*: ST18, ST64 and ST767 were found only in Type I, while ST341 was only in Type II.The remaining 6 structure types were sporadically found in 1–3 isolates, respectively (Fig. 3). The insertion of IS*1251*-like found in Types I and II were also found in Types III∼VI, making the insertion of IS*1251*-like the most predominant insertion sequences (128 isolates, 97.0%) among the isolates studied. The extra insertion of IS*1678* found in Type II also could be found in Types III and VI (65 isolates, 49.2%). Large truncations in the regions consisting of *orf1*, *orf2* and *vanR* and/or those consisting of *vanX*,*vanY*, or *vanZ* were found in Types IV, VII and VIII, as no amplicons could be obtained by the associated primer pairs.

The association of various structure types of Tn*1546* with the major pulsotypes A–G was different. Pulsotypes A, B, and G were all associated with Type I, while Type II was found in all pulsotypes D and E isolates and prevalent in pulsotypes C and F. In pulsotype F, the five isolates from the northern region all carried the Type II Tn*1546*. Accordingly, among the 5 genotype clusters found in hospitals C_1_, N_1_ and N_2_, ST17/pulsotype A, ST17/pulsotype B, and ST18/pulsotype G were all associated with Type I, while ST341/pulsotype D and ST414/pulsotype E were associated with Type II.

## Discussion

A total of 13 STs were identified in the present study. All of them belonged to CC17, the most frequently identified clonal complex globally [28]. CC17 is characterized by the multi-drug resistance to ampicillin and fluoroquinolones and is a globally distributed nosocomial-related lineage [9], [29]. Among them, three STs, ST17, ST414 and ST78, were predominant during the study period and could be identified from different regions in Taiwan. ST17 and ST78 were the worldwide spreading epidemic clones, and have been identified among hospitalized patients in almost all continents, including Asia, North and South America, Europe, and Australia [11], [30]–[33]. In contrast, ST414 (16.4%) was the second most prevalent ST in the present study but was less common in other countries. In fact, it was firstly identified from Australia in 2008 [34]. Later, recent reports from Asia, including China [6], [35], Hong Kong [36], and Taiwan [15], [19], also demonstrated the emergence of ST414. According to a recent outbreak investigation from northern Taiwan, the proportion of ST414 could be as high as close to 50% [19]. However, up to the present, ST414 has not yet been reported from the western countries. Whether or not this newly emergent Asia clone, ST414, will soon be discovered or widespread in western countries warrants close monitoring.

Vancomycin resistance is due to the presence of *van* genes, including *van*A, *van*B, and other minor *van* genes [10]. In the present study, all but two isolates carried the *van*A gene. The results were consistent with reports from other countries [4], [37]. However, the proportions of *van*B-containing isolates in these studies were about 10%–20%, much higher than the <2% found in the present study. The results suggested that the increase of VRE-fm isolates in Taiwan may be related to the spread of the *van*A gene. Furthermore, previous reports from Taiwan demonstrated a high prevalence (51%–90%) of VRE isolates showing a specific VanB phenotype-*van*A genotype [7], [14], [22]. In the present study, however, the proportion of such isolates greatly reduced to 2.3%. Reports from other countries remained rather constant at 5%–12% [6], [33]. Therefore, the decrease of such specific *van*A genotype -VanB phenotype among the VRE isolates in Taiwan as presented in this study remains to be elucidated.

The *van*A gene is known to be carried by Tn*1546* [25]. Several genetic arrangements of the Tn*1546* have been reported [8], [11], [12]. In the present study, we also identified eight structure types of Tn*1546*, with Types I and II being the most prevalent. Type I is characterized by the insertion of IS*1251*-like, which could also be found in Types II–VI. Actually, only four (3.0%) of our study isolates did not have this insertion. Insertion of IS*1251* in the Tn*1546* was firstly described in the United States in 1995 [38]. In 1999, Willems et al. reported a Tn*1546* F1 Type [12], in which the IS*1251* was also inserted between *vanS* and *vanH* genes, similar to our Type I structure. Recent reports indicated that such a structure was not only prevalent in Taiwan (36.7%), but also in Brazil (87.8%), and Paraguay (92.5%) [11], [30], [32]. Type II is characterized by an extra insertion of IS*1678* between *orf2*-*vanS* genes. IS*1678* was only recently reported in Korea with a different insertion site at the intergenic region between *vanX* and *vanY* genes [39]. Therefore, the finding that almost a half of the VRE-fm isolates studied herein carried a Tn*1546* of this structure type is surprising. Moreover, according to our preliminary data on the 152 VRE isolates collected in 2013, the proportion of the isolates carrying this Type II Tn*1546* has increased to ∼60%. The mechanism underlying the emerging and widespread of the Type II Tn*1546* warrants further studies.

Another characteristic of the present study was that the left extremity of Tn*1546* was lost in all isolates studied. Several reports also demonstrated similar findings [11], [12], [30], [32], [40]. This region corresponded to gene *orf1* which was associated with transposition functions [10]. A previous study indicated that this truncated Tn*1546*-like element plus the insertion of IS*1251*-like was associated with a much higher conjugation frequency compared to that shown by the prototype Tn*1546* (10^−6^ vs. 7×10^−7^) [11]. It seems that such a structural change (truncated *orf1* plus insertion of IS*1251*-like) in Tn*1546* may facilitate the dissemination of the transposon, so as that of the *van* gene cassette. It may also explain the finding that the majority (95.5%) of the isolates studied herein carried a Tn*1546*-like element that consisted of such a changed structure. The less dissemination ability of the prototype Tn*1546* may lead to the decrease of its prevalence. Therefore, similar to a recent study [41], we could not find the prototype in any of our isolates studied.

In conclusion, our results revealed highly diverse genotypes among the VRE clinical isolates in Taiwan. However, we also found the predominance of three major ST types (ST17, ST414, ST78), seven predominant pulsotypes, and two prevailing structure types of the Tn*1546-*like element (Types I and II) from different hospitals and regions in Taiwan. Therefore, both the horizontal transfer of *van*A genes through some efficient Tn*1546-*like elements and the clonal dissemination of some major genotypes within or among the hospitals may have contributed the overall increase of VRE in Taiwan. Furthermore, mechanisms underlying the high proportion of ST414 and isolates carrying the Type II Tn*1546*-like elements may warrant a further study.
